# Daidzein Synergizes with Gefitinib to Induce ROS/JNK/c-Jun Activation and Inhibit EGFR-STAT/AKT/ERK Pathways to enhance Lung Adenocarcinoma cells chemosensitivity

**DOI:** 10.7150/ijbs.71870

**Published:** 2022-05-16

**Authors:** Thomas Gabriel Mhone, Ming-Cheng Chen, Chia-Hua Kuo, Tzu-Ching Shih, Chung-Min Yeh, Tso-Fu Wang, Ray-Jade Chen, Yu-Chun Chang, Wei-Wen Kuo, Chih-Yang Huang

**Affiliations:** 1 Graduate Institute of Biomedical Sciences, China Medical University, Taichung 404, Taiwan; 2 Division of Colorectal Surgery, Department of Surgery, Taichung Veterans General Hospital, Taichung 40705, Taiwan; 3 Faculty of Medicine, National Yang-Ming University, Taipei, Taiwan; 4 Laboratory of Exercise Biochemistry, University of Taipei, Taipei, Taiwan; 5 Department of Biomedical Imaging & Radiological Science College of Medicine, China Medical University, Taichung 404, Taiwan; 6 Department of Pathology, Changhua Christian Hospital, Changhua 500, Taiwan; 7 Department of Hematology and Oncology, Hualien Tzu Chi Hospital, Buddhist Tzu Chi Medical Foundation, Hualien, Taiwan; 8 School of Medicine Tzu Chi University, 701, Section 3, Chung-Yang Road, Hualien 97004, Taiwan; 9 Department of Surgery, School of Medicine, College of Medicine, Taipei Medical University, Taipei 11031, Taiwan; 10 Cardiovascular and Mitochondrial Related Disease Research Center, Hualien Tzu Chi Hospital, Buddhist Tzu Chi Medical Foundation, Hualien, Taiwan; 11 Department of Biological Science and Technology, China Medical University, Taichung 406, Taiwan; 12 Ph.D. Program for Biotechnology Industry, China Medical University, Taichung 406, Taiwan; 13 Center of General Education, Buddhist Tzu Chi Medical Foundation, Tzu Chi University of Science and Technology, Hualien 970, Taiwan; 14 Department of Medical Research, China Medical University Hospital, China Medical University, Taichung 404, Taiwan; 15 Department of Biotechnology, Asia University, Taichung 413, Taiwan

**Keywords:** Chemosensitivity, Daidzein, ROS, Synergistic, c-Jun, Apoptosis

## Abstract

Lung cancer is the major cause of cancer associated mortality. Mutations in EGFR have been implicated in lung cancer pathogenesis. Gefitinib (GF) is a RTKI (receptor tyrosine kinase inhibitor) first-choice drug for EGFR mutated advanced lung cancer. However, drug toxicity and cancer cell resistance lead to treatment failure. Consequently, new therapeutic strategies are urgently required. Therefore, this study was aimed at identifying tumor suppressive compounds that can synergistically improve Gefitinib chemosensitivity in the lung cancer treatment. Medicinal plants offer a vast platform for the development of novel anticancer agents. Daidzein (DZ) is an isoflavone compound extracted from soy plants and has been shown to possess many medicinal benefits. The anticancer potential of GF and DZ combination treatment was investigated using MTT, western blot, fluorescent microscopy imaging, flow cytometry and nude mice tumor xenograft techniques. Our results demonstrate that DZ synergistically induces c-Jun nuclear translocation through ROS/ASK1/JNK and downregulates EGFR-STAT/AKT/ERK pathways to activate apoptosis and a G0/G1 phase cell cycle blockade. In in-vivo, the combination treatment significantly suppressed A549 lung cancer cells tumor xenograft growth without noticeable toxicity. Daidzein supplements with current chemotherapeutic agents may well be an alternative strategy to improve the treatment efficacy of lung adenocarcinoma.

## Introduction

Lung cancer is leading type of cancer and globally and the commonest cause of cancer associated death 1. Lung adenocarcinoma accounts for 40% lung cancer cases. Majority of lung cancer patients display advanced disease at diagnosis heavily contributing a poor prognosis and a low 5-year overall survival 2, 3. EGFR activating mutations has been implicated in pathogenesis of lung cancer 4. Gefitinib (GF) is the first-choice drug for EGFR mutated lung cancer 5. However, treatment failure due to drug toxicity and drug resistance has been reported under 15 months of chemotherapy 6. Consequently, new therapeutic strategies devoid of toxicity are urgently required.

Physiological ratios of reactive oxygen species (ROS) and antioxidants is tightly regulated. ROS disequilibration leads to DNA and other cellular components damage, activating cell death processes. Substantial research evidence has shown that medicinal plants derived compounds can instigate cell death by ROS persistent generation 7. ASK1/MAPK signaling pathway has been shown to be activated by prolonged ROS activity. Once activated ASK1 induces apoptic cell death through the stress activated JNK/SAPK and p38 MAPK pathways 8. Activated JNK consequently translocate to the mitochondrial membrane to downregulate Bcl-2 and release cytochrome c to induce apoptosis.

Daidzein (DZ) is an isoflavone compound extracted from soy plants and it has been shown to possess many medicinal benefits. Previous preclinical studies have demonstrated Daidzein's antiproliferative effects on numerous cancer cells 9-15. Here we show that Daidzein synergizes with Gefitinib via ROS mediated c-Jun nuclear translocation and suppresses EGFR-STAT/AKT/ERK signalling axis to induce a G0/G1cell cycle arrest and apoptosis in A549 and H1975 lung adenocarcinoma cells.

## Materials and Methods

### Cell culture

A549, BEAS-2B, H9C2 and Clone 9 cells were maintained at 37 ^0^c in DMEM medium (D5523, Sigma,). H1975 and LoVo cancer cells were cultured in RPMI-1640 (Gibco). 10% Fetal Bovine Serum (Characterized FBS, Hyclone, long, UT) and 1% penicillin/streptomycin (Invitrogen Corp) was supplemented to all culture media, in a 5% CO_2_ enriched environment.

### Drugs

Daidzein (Cas no. 486-66-8) and Gefitinib (CAS No. 184475-35-2) were purchased from Cayman Chemical 1180 East Ellsworth Road Ann Arbor, Michigan 48108 USA. NSC 228155 EGFR Activator (CAS No. 113104-25-9) was purchased from MedChemExpress LLC, 1 Deer Park Dr, Suite Q, Monmouth Junction, NJ 08852, USA. JNK inhibitor SP600125 (CAS No. 129-56-6) and NAC (N-Acetyl-L-cysteine (CAS Number: 616-91-1) was purchased from Sigma-Aldrich, 3050 Spruce St. Saint Louis, MO, 63103-2530, United States.

### Cell Viability MTT assay

The viability of all tested cells was determined by MTT [3‐(4, 5‐Dimethylthiazol‐2‐yl) ‐2, 5‐diphenyltetrazolium‐bromide] (Sigma). Cells were seeded in triplicates in a 24‐well plate and treated with grading concentrations of Gefitinib (10, 20, and 30 μM) and Daidzein (100, 200, and 300 μM) for 24 and 48 hours followed by MTT for 2-4 hours (0.5 mg/ml final concentration). DMSO was used to dissolve the reduced purple MTT formazan crystals. Color intensity absorbance of controls vs treated groups was readout at 595 nm in an ELISA reader. Viability was expressed as proportion of dead cells in treatment groups versus the control group. For the combination treatment, Gefitinib (10 μM or 30 μM) was first treated for 24 h followed by Daidzein (300 μM) without changing media for an additional 24 h followed by MTT 16. We used ComboSyn software to calculate combination index (CI). In our study, CI > 1 indicates antagonism, CI = 1 additive, and CI < 1 synergism 16, 17.

### Whole cell lysate and nuclear protein extract

Lysis buffer (1 mM Tris‐base, 5 M NaCl, 0.5 M EDTA, 1% NP40, 1mM EDTA, Protease inhibitor and phosphatase inhibitor) was used to extract total proteins from the cells as previously described 18. The collected total proteins were stored at -80°C. Nuclear protein extraction kit (BioVision) was used to fractionate nuclear and cytosol proteins according to the kit's protocol. Briefly, trypsinized cells were centrifuged, followed by addition cytosol extraction buffer mix contain protease inhibitor and DTT (CEB-A). Then cytosol fraction was collected after adding cytosol extraction buffer B (CEB-B) and centrifugation. Nuclear extraction buffer mix (NEB) containing protease inhibitor and DTT was added to the sediment pellet and incubated on ice, vortexing every 10 minutes for 40 minutes followed by centrifugation. The collected supernatant was stored at -80 ^o^ c.

### Bradford Protein Assay

Bradford Protein assay was used to determine protein concentration. Briefly, 20 μl of protein samples and serially diluted 1mg/ml Bovine Serum Albumin (BSA) standards were incubated in Coomassie protein blue dye. Absorbance of protein-dye complex was read out at 595 nm in an ELISA reader. A standard curve was constructed from the absorbances of the standards which was then used to estimate the concentration of protein in the samples 18, 19.

### Western blotting

Equalized amounts of whole cell lysates and extracted nuclear protein (30 -50 µg) quantified as above were heated in 5x sample buffer for 5-10 minutes. The samples were separated on 7-15% SDS-polyacrylamide gels (PAGE) and transferred onto Polyvinylidene fluoride (PVDF) membrane. Then the membranes were blocked with 5% nonfat milk followed by overnight staining with specific primary antibody at 4°C and finally secondary antibody for 1 hr at room temperature as previously described 20, 21

### Determination of ROS production

CM-H2DCFDA General Oxidative Stress Indicator (Invitrogen) and MitoSOX™ Red (Invitrogen) stains were used to determine cellular ROS and mitochondrial generated ROS respectively. The experiments were performed according to kit's protocol. Briefly, CM-H2DCFDA in PBS (10 µM concentration) was incubated in A549 cells seeded on chamber slide for 1 hr, followed by fluorescence microscopy. For mitochondria ROS, cells were incubated for 30 minutes in MitoSOX Red in PBS (5 µM concentration), followed by fixation (4% paraformaldehyde) and permeabilisation (0.2% Triton X). Finally, drops of Fluoroshield with DAPI counter stain were added before analysis under fluorescence microscope.

### TUNEL green and Tunel red Staining

In situ Cell death Detection Kit, TMR red and Fluorescein kits (Roche) were used to stain apoptotic positive cells according to kit's manual. Briefly, A549 cancer cells grown on chamber slides were fixed with 4% formalin solution for 30 minutes, and then permeabilized with 0.2% Triton X for 2 minutes on ice. Next, the cells were stain with Tunel in the dark for one hour at 37°C. Finally, Fluoroshield with DAPI counter stain was added to the slides and sealed with cover glass before fluorescence microscopy 22.

### Annexin-V/PI double staining Flow Cytometry

Annexin-V and PI double staining (BD Biosciences) apoptosis detection was performed according to kit's protocol. Briefly, approximately (1 × 10^6^ cells/dish) A549 cancer cells seeded in a 10 cm dish were trypsinized and collected by centrifugation, then staining buffer was added (containing Annexin V-FITC and propidium iodide in binding buffer) followed by flow cytometry analysis (BD FACSCanto II). The apoptosis rate (percentage of dead cells compared to normal cells) for each treatment group was obtained 23.

### PI Staining Cell Cycle

Briefly, treated and untreated A549 cancer cells were trypsinized and fixed with cold 75% ethanol for 30 minutes or over‐night at -20°C. Ethanol was removed and the cells stained with staining solution (containing 50 µg/ml PI, and 100 µg/ml RNaseA in PBS), followed by analysis and quantification by BD FACSCanto II flow cytometer, as previously described 24.

### Immunofluorescence imaging

Cells were fixed in 4% formalin, and then permeabilized in 0.2% Triton X-100, then blocked with 10% Goat Serum for 1 hour. Consequently, primary antibody (p-c-Jun; 1:500 in 1% goat serum) was overnight incubated at 4^o^c. Next, the Alexa Fluor 488 goat anti-rabbit secondary antibody (1:500 in 1% goat serum) was incubated for 1 h at room temperature and followed by Fluoroshield DAPI mounting medium and microscopy.

### Mouse tumor xenograft study

Male NU/NU nude mice (six-week-old) were procured from BioLASCO Taiwan Co., Ltd. (Taipei, Taiwan) and cared for under the guidelines of the Laboratory Animal Service Center (LASC) of China Medical University (CMU) in compliance with the principles of the 3Rs (Replacement, Reduction and Refinement) and Humane Care and Use of Laboratory Animals policy. The animal use protocol described below was reviewed and approved by LASC CMU Committee (Approval code no. 2021-253). Briefly, 4 × 10^6^ A549 cancer cells in Matrigel serum free medium (BD Biosciences) suspension were subcutaneously injected into 8-week-old male NU/NU mice hind legs. A digital caliper was used to measure tumor height, length and width, and the volume was calculated with a modified ellipsoidal formula: 1/2 × (length × width × thickness) as previously described 25. When the tumor sizes reached 500 mm^3^ the mice were divided into four treatment groups, three animals per group. The mice were treated twice a week for five weeks through oral gavage. The mice were fed with either Gefitinib 100 mg/kg or Daidzein 300 mg/kg and the combination of the two in canola oil. The control group were given only canola oil. The mice were euthanized at the experimental endpoint and their tumors and organs removed. Small fresh cut pieces of tumor tissue and internal organs were stored at -80^o^C and the rest were fixed in 10% formalin embedded in paraffin. Serum was separated from the collected whole blood and stored at -80^o^C.

### Toxicity evaluation and human to animal drug dose conversion

To check animal welfare, body weights weekly measurement was done. Additionally, we twice a week checked the health-welfare of each animal by observing their general appearance, feeding behavior, breathing and movement. We observed as well for pathological signs such as bleeding and diarrhea. After sacrificing the animals at the end of treatment period, gross examination of internal organs was conducted. Blood serum was analysed for liver and kidney drug induced toxicity. To calculate the human to animal drug doses we used the formula based on body surface area (Human dose (mg / kg) = Animal dose (mg / kg) × Km ratio) as previously described by 26, 27. We reduced Gefitinib clinical dose 2.5-fold to reduce the toxicity.

### Survival curve for in vivo tumors

Kaplan-Meier method was used to evaluate animal survival curves. The mice were considered expired once the tumor size reached 1,500 mm^3^ during the treatment period, as previously described 28, 29.

### Immunohistochemistry

Tissue slides were deparaffinized by Xylene and rehydrated with serially degrading ethanol, next, the slides were immersed in citric acid for antigen retrieval. 0.3% hydrogen peroxide was used to inhibit endogenic peroxidase activity. 10% normal goat serum was used to block the sections for 30 minutes. Primary antibody (p-c-Jun dilution, 1:500) was incubated overnight at 4^o^C. Next biotinylated secondary antibodies and streptavidin-biotin complex (Lab Vision, Fremont, CA) was added and incubated for 1 hour. Finally, DABI substrate was added and the slides were dehydrated with serially increasing ethanol and xylene.

### Statistical analysis

Variations between multiple groups were calculated using ANOVA with appropriate post-hoc tests except where other methods mentioned. p-values of < 0.05 were considered statistically significant. All experimentations were performed in triplicates.

## Results

### Daidzein Effect on normal and cancer cells

**Figure 1:**
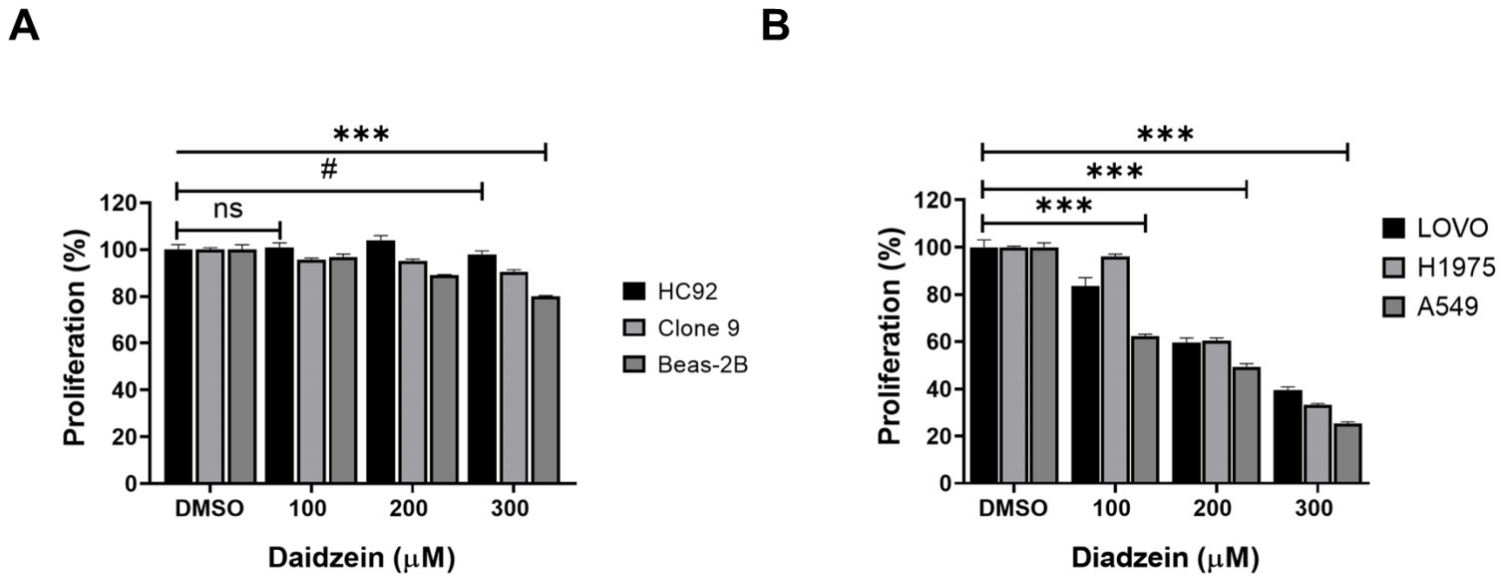
** Daidzein Effect on normal and cancer cells.** (**A**) The effect of Daidzein on normal heart (H9C2), liver (Clone 9) and lung cells (Beas-2B) cells, cell viability was evaluated by MTT assay. (**B**) The influence of Daidzein on LoVo (colorectal) cancer cells, H1975 and A549 (lung adenocarcinoma) cells when treated with indicated concentrations after 24 h. These results are expressed as a percentage of viable cells from treated groups compared with control cells treated with DMSO The results are shown as means ± standard deviation from three independent experiments. ns: not significant, # p < 0.05, *** p < 0.0001.

We firstly evaluated the toxicity of Daidzein in normal heart H9C2 cells, liver Clone 9 cells, lung Beas-2B cells using MTT cell viability assay. Different concentrations of Daidzein (0, 100, 200 and 300 µM) were treated to cells for 24 h. The results indicate that Daidzein at these concentrations is relatively nontoxic to the treated normal cells (**Figure 1A**). We next investigated the cytotoxic effect of Daidzein against A549, H1975, and LOVO cancer cells. Our results demonstrates that Daidzein significantly reduced proliferation of the tested cells dose dependently at 24 h (**Figure 1B**) with IC_50_ of 226.2 µM ±1.2 in A549 cancer cells, 257.3 µM ±1.1 in H1975 cancer cells, and 249.2 µM ±1.3 in LOVO cancer cells (**Table 1**). These results suggest Daidzein may be used as a complementary treatment to improve the chemosensitivity of cancer cells.

### Daidzein and Gefitinib *c*ombination treatment synergistically inhibits Lung adenocarcinoma cells viability

**Figure 2:**
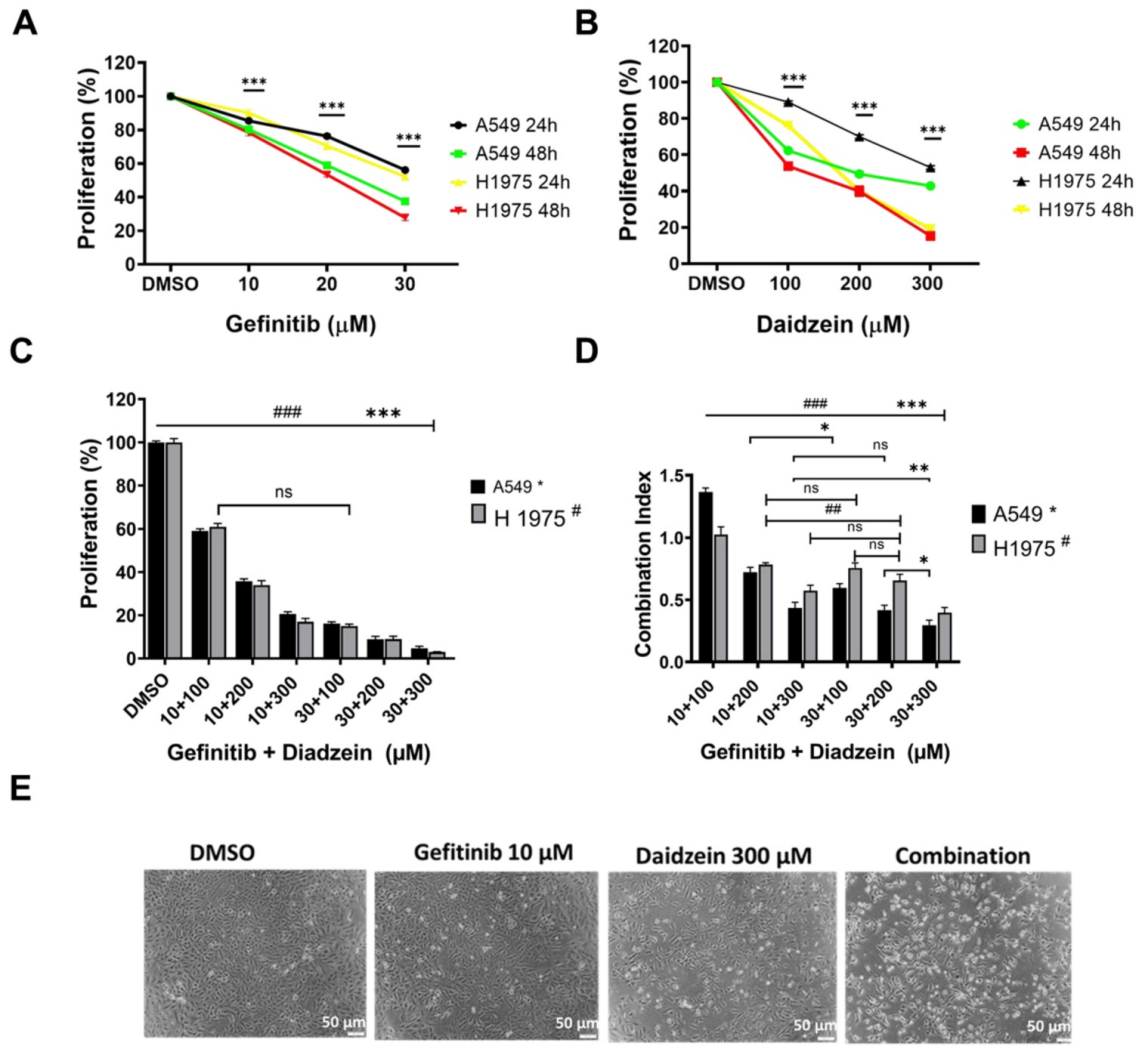
** Daidzein and Gefitinib combination treatment synergistically inhibits Lung adenocarcinoma cells cell viability.** (**A**) A549 and H1975 cancer cells were treated with indicated concentrations of Gefitinib for 24 and 48 hours and then their cell viabilities were determined by MTT assay. (**B**) As in A, treated with indicated concentrations of Daidzein. (**C**) Evaluation of synergistic potential of Daidzein and Gefitinib combination treatment, A549 and H1975 cancer cells were co-treated with either 10 µM or 30 µM of Gefitinib and the indicated concentrations of Daidzein followed by MTT assay cell viability evaluation. (**D**) Calculation of the combination index (CI) of data in figure B. The CI reflects the degree of drug-drug interactions; in this study, CIs <0.9 indicated synergism, ranging from 0.9 to 1.1, additive and >1.1, antagonistic effects. These results are expressed as a percentage of viable cells from treated groups compared with control cells treated with DMSO. (**E**) Phase contrast microscopy depicting morphological changes in A549 cancer cells when treated with the indicated drugs and concentrations. Representative images are shown. Scale bars: 50 μm. Results are shown as means ± standard deviation from three independent experiments. ns: not significant, * p< 0.05, ## <0.001, ###; *** p < 0.0001.

Combination treatment has been demonstrated to improve cancer cell chemosensitivity through various mechanisms 30. Gefitinib is first generation drug for advanced lung cancer with a positive EGFR mutation. The recommend clinical daily oral dose of Gefitinib (250 - 500 mg/kg) has been reported to induce complications, and secondary EGFR mutations contributes to treatment failure 31. Daidzein has been reported to possess many antitumor effects, and we have demonstrated it to be relatively nontoxic to normal cells (**Figure 1A**). We therefore hypothesized that lowering the daily dose of gefitinib, supplemented with Daidzein would synergistically chemosensitize lung cancer cells to reduce toxicity in NSCLC. We therefore challenged A549 and H1975 lung adenocarcinoma cell lines with Daidzein alone or in combination with Gefitinib and the cytotoxicity was assessed by MTT assay. Both compounds demonstrated a time and dose dependence growth inhibition of A549 and H1975 lung cancer. The IC_50_ of Gefitinib in A549 cancer cells was 22.8 µM ±0.4 and H1975 cancer cells was 21.7 µM ±1.0 at 48 h respectively. Daidzein IC_50_ in A549 cancer cells were 226.2 ±1.2 and 130.5 ±1.6 µM at 24 and 48 h respectively. Meanwhile, Daidzein IC_50_ in H1975 cancer cells were 257.3 µM ±1.1 and 186.5 µM ± 1.1 at 24 and 48 h respectively (**Table 1**)**,** (**Figure 2A**, and** B**). To show that low doses of Gefitinib supplemented with Daidzein would have synergistic cytotoxicity, we first sensitized A549 and H1975 cancer cells with Gefitinib and then Daidzein was added after 24 h for a combined 48 h treatment period. Indeed, our results revealed that Daidzein significantly enhanced Gefitinib chemosensitivity inhibiting proliferation of both cell lines (**Figure 2C**). All the combination pairs exhibited synergistic effects (except for 10 µM Gefitinib + 100 µM Daidzein) (**Figure 2D**). We therefore selected a low dose Gefitinib (10 µM) and high dose Daidzein (300 µM) for all our subsequent experiments since these doses demonstrated a strong synergism (CI value < 0.7), and a 300 µM dose of Daidzein demonstrated to be nontoxic when tested in normal cells (**Figure 1A**). Consequently, phase contrast microscopy confirmed that our selected combination doses enhanced morphological changes in A549 cancer cells more than the single drugs alone (**Figure 2E**). Taken together, these data demonstrates that Daidzein synergistically enhances Gefitinib cytotoxicity. Daidzein supplements would improve chemotherapeutic responses in patients taking Gefitinib for lung adenocarcinoma.

### Gefitinib and Daidzein Combination Treatment Induces ROS Mediated Cell Death

**Figure 3:**
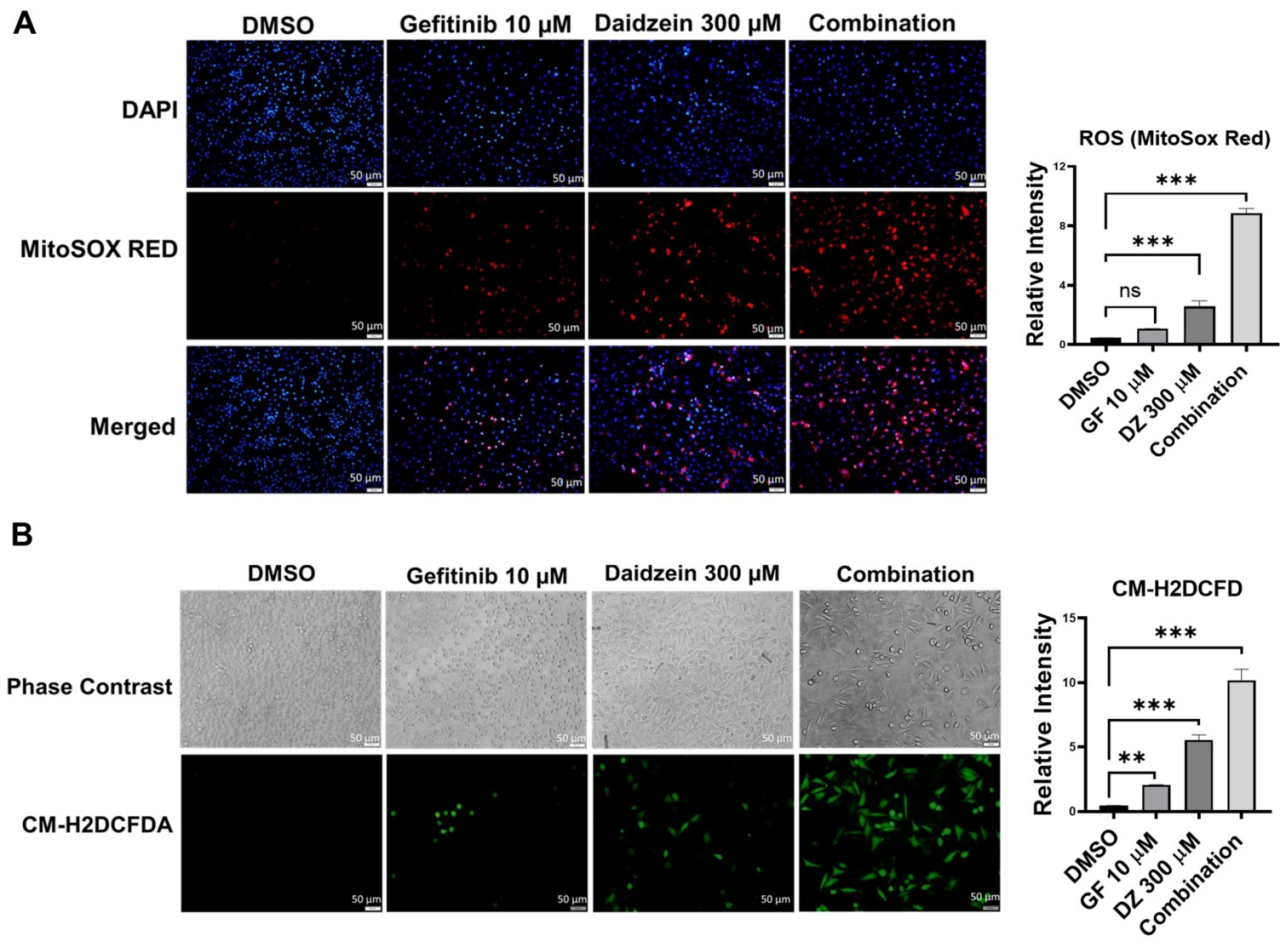
** Daidzein and Gefitinib Combination Treatment Induces ROS Mediated Cell Death.** ROS Immunofluorescence Analysis and quantification. A549 cancer cells were treated with either Gefitinib (10µM), or Daidzein (300µM), and combination of the two drugs followed by staining. (**A**) MitoSOX Red staining and quantification demonstrating mitochondrial generated ROS, nuclei were counterstained with DAPI mounting medium. (**B**) CM-H2DCFDA general cellular ROS indicator and quantification. Results are shown as means ± standard deviation from three independent experiments. Representative images are shown. Scale bars: 50 μm. ns: not significant, ** p < 0.001, *** p < 0.0001.

ROS has been revealed to mediate in many metabolic processes in health and disease. Dysregulation of ROS induces cellular damage and can activate both intrinsic and extrinsic apoptotic cell death 32. Previously Daidzein has been shown to induce ROS in different cell lines 34-36. To establish whether Gefitinib and Daidzein combination treatment can synergistically stimulate mitochondrial oxidative stress, we investigated the mitochondrial generated ROS levels using MitoSOX Red fluorescence staining, a specific fluorogenic dye that is rapidly oxidized by superoxide in the mitochondria of live cells. Our results revealed that Daidzein and Gefitinib combination treatment significantly enhanced mitochondrial ROS generation in A549 cells compared to single drugs alone (**Figure 3A**). To distinguish between mitochondrial generated ROS and general ROS we used CM-H2DCFDA staining a general cellular ROS indicator in live cells. CM-H2DCFDA is changed to a greatly fluorescent 2',7'-dichlorofluorescein (DCF) upon ROS oxidation. Similarly, Daidzein and Gefitinib combination treatment significantly induced increased production cellular ROS compared to the single drugs alone (**Figure 3B**). Together, these data suggest that Daidzein and Gefitinib combination treatment cell proliferation inhibition may be induced by increased production of ROS.

### Gefitinib and Daidzein Combination Induces both Intrinsic and Extrinsic Apoptosis

**Figure 4:**
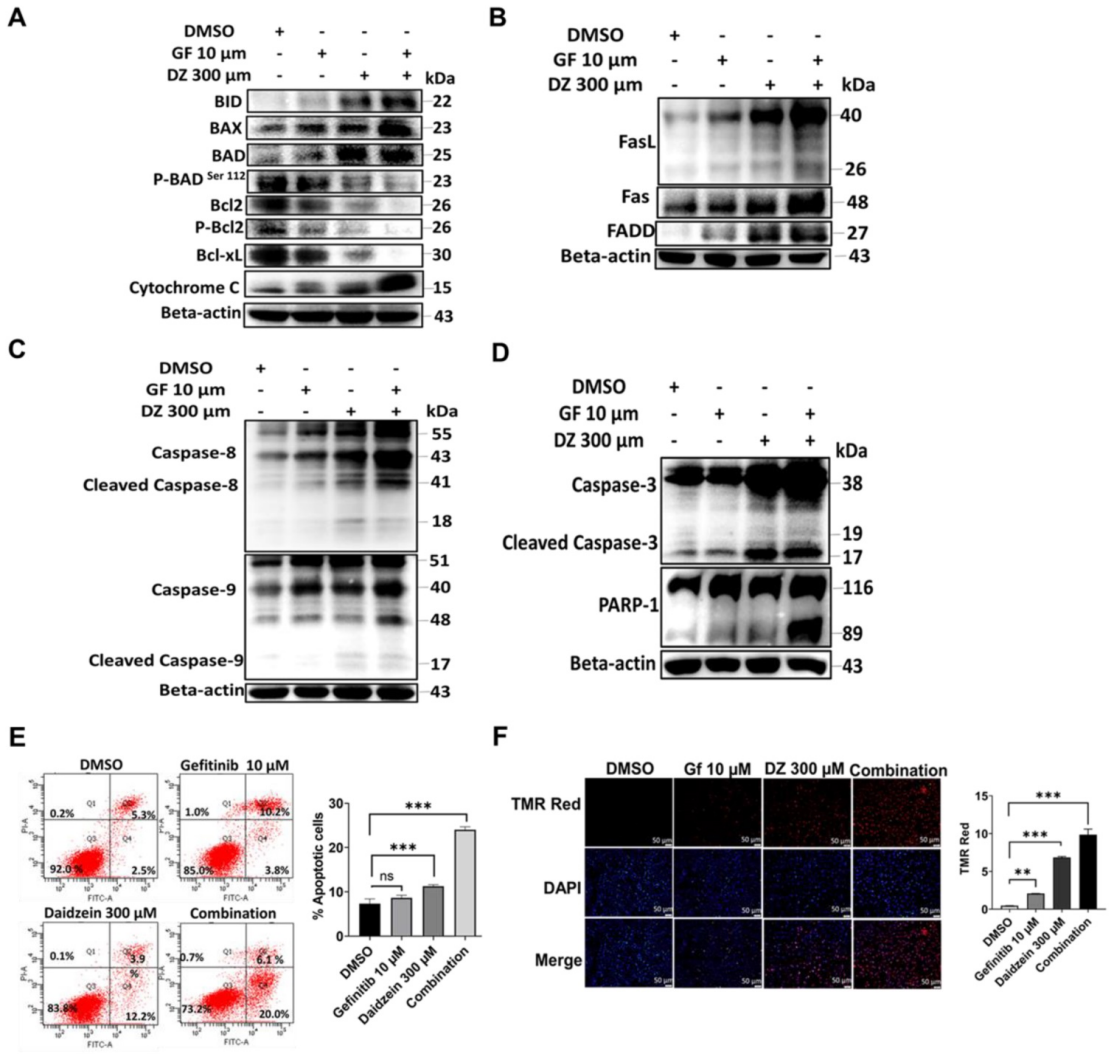
** Daidzein and Gefitinib Combination synergistically enhances both Intrinsic and Extrinsic Apoptosis.** Immunoblotting of apoptotic markers in A549 cancer cells whole cell lysates treated with either Gefitinib (10µM), or Daidzein (300µM), and combination of the two drugs (**A**) Activation of intrinsic apoptosis (**B**) Activation of extrinsic apoptosis (**C**) Activation of caspases 8 and 9 (**D**) Activation of caspases 3 and PARP-1. Beta-actin is used as the loading control. (**E**) Annexin V-FITC and PI staining of apoptotic positive cells flow cytometric analysis and quantification, same treatment as in A. (**F**) TUNEL TMR red staining of apoptotic positive cells and quantification. Nuclei were stained by DAPI mounting medium. Representative images are shown. Scale bars: 50 μm. Results are shown as means ± standard deviation from three independent experiments, ns: not significant, **P<0.001, ***P<0.0001.

Apoptosis can be triggered by a diversity of stress signals such as ROS stress 37. Our data in **Figure 3** confirmed that Daidzein and Gefitinib combination treatment induces ROS. We next evaluated whether ROS induced by our combination treatment led to activation intrinsic and extrinsic apoptosis. We evaluated the expression levels of apoptotic markers through western blot. As expected, combination treatment significantly activated intrinsic apoptosis through activation of Bad, Bax, and Bid and reduction of Bcl2 and Bcl-xL and release of Cytochrome C (**Figure 4A**). Similarly, extrinsic apoptosis was confirmed by activation of FasL, FAS and FADD (**Figure 4B**). Activation of intrinsic and extrinsic apoptosis led to cleaving of caspase 8 and 9 and activation (**Figure 4C**) and finally cleaving of caspase 3 and PARP1 activation (**Figure 4D**). Apoptotic cell death was confirmed by flow cytometric analysis of Annexin V-FITC and PI positive staining apoptotic cells (**Figure 4E**). Additionally, apoptosis was confirmed by increasing number of Tunel positive cells in the combination treatment group when stained with In Situ Cell Death Detection Kit, TMR red (**Figure 4F**). Together, these data demonstrates that Gefitinib and Daidzein combination treatment synergistically induced apoptosis in A549 and H1975 lung cancer cells.

### NAC Reverses Daidzein and Gefitinib Combination treatment induced ROS Mediated Cell Death

**Figure 5:**
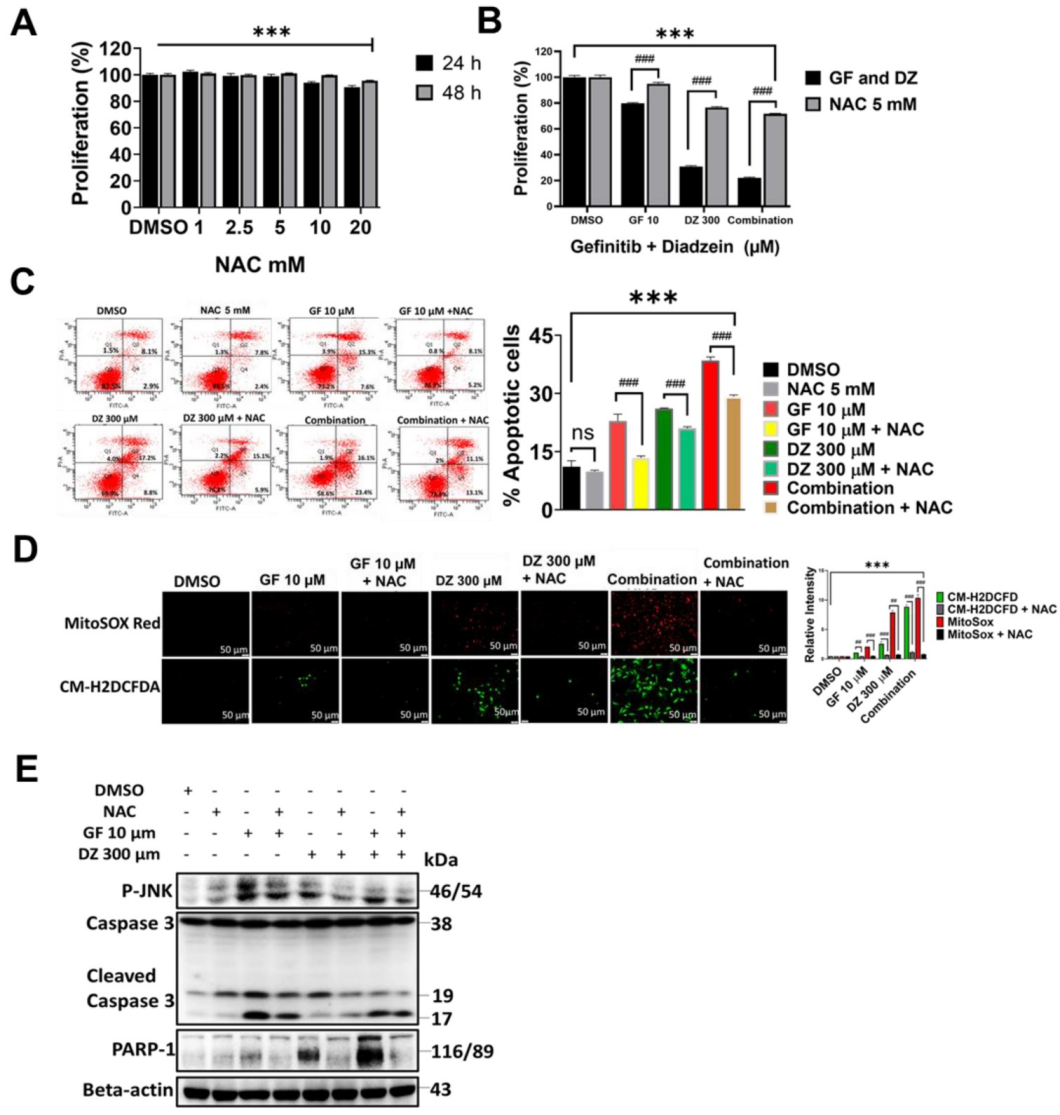
** NAC Reverses Daidzein and Gefitinib Combination treatment induced ROS Mediated Cell Death.** (**A**) Influence of NAC on A549 cancer cells viability, different concentrations of NAC (1 - 20 mm) were treated to A549 cancer cells followed by MTT cell viability assay. (**B**) NAC reverses Daidzein and Gefitinib Combination treatment induced cell death. A549 cancer cells were treated with either Gefitinib (10 µM) or Daidzein (300 µM) and combination of the two, with or without NAC (5mM) followed by MTT cell viability assay. (**C**) Treatment as in B, followed by Annexin V-FITC and PI staining of apoptotic positive cells, flow cytometric analysis and quantification. (**D**) As in B, MitoSOX and CM-H2DCFDA staining and quantification. Representative images are shown. Scale bars: 50 μm. (**E**) Treatment as above, immunoblotting of P-JNK, Caspase 3 and PARP-1 in A549 cancer cells whole cell lysates. Beta-actin is the loading control. Results are shown as means ± standard deviation from three independent experiments. ns: not significant, ## P<0.001, ###; *** P<0.0001.

NAC is a potent ROS inhibitor and has been previously demonstrated to reverse drug induced oxidative stress 38. To further evaluate the ROS activation and the cell death due to Daidzein and Gefitinib combination treatment, we investigated the effects of co-treatment with NAC antioxidant. We identified 5 mM as a NAC working dose (**Figure 5A**). Co-treatment of NAC dramatically reversed the cell viability loss induced by Daidzein and Gefitinib combination treatment (**Figure 5 B**). Annexin V-FITC and PI staining cytometric analysis revealed that co-treatment with NAC significantly decreased combination treatment induced apoptosis (**Figure 5 C**). Furthermore, MitoSOX Red and CM-H2DCFDA staining demonstrated that NAC effectively blocked combination treatment induced ROS (**Figure 5D**). Western blot analysis revealed that NAC reversed Daidzein and Gefitinib combination treatment induced activation of apoptosis-related proteins (**Figure 5E**). These results taken together reveal that apoptosis triggered by combination treatment is associated with ROS generation.

### Daidzein Synergizes with Gefitinib to inhibits EGFR/STAT/AKT/ERK in Lung Adenocarcinoma cells

**Figure 6:**
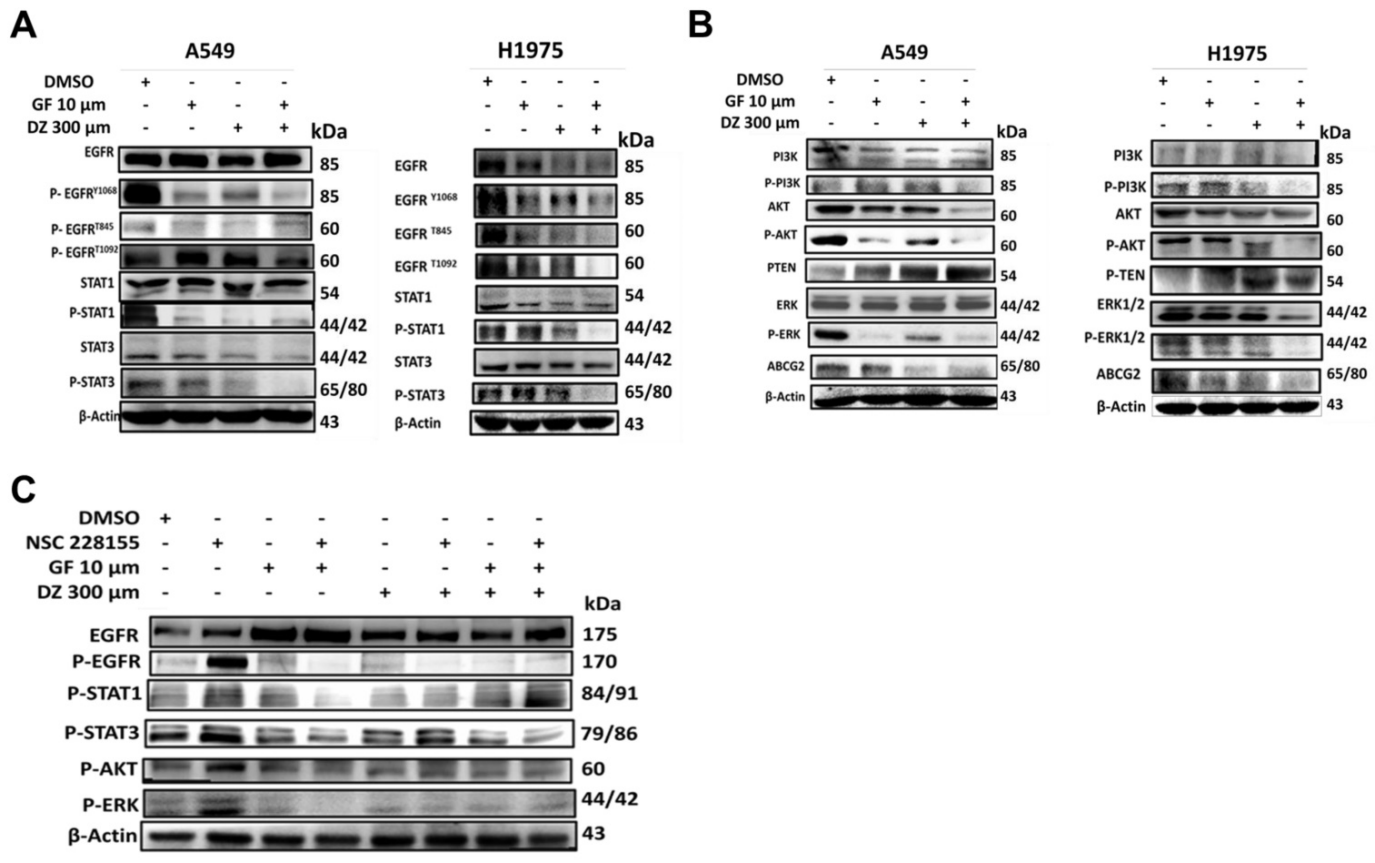
** Daidzein Synergizes with Gefitinib to inhibit EGFR/STAT/AKT/ERK and in Lung Adenocarcinoma cells.** Immunoblotting of A549 and H1975 cancer cells whole cell lysates treated with either Gefitinib (10µM), or Daidzein (300µM), and combination of the two drugs. (**A**) Inhibition of EGFR/STAT, (**B**) Inhibition of AKT/ERK pathways, (**C**) Confirmation of EGFR inhibition, immunoblotting of A549 cancer cells whole cell lysates treated with Gefitinib (10µM), or Daidzein (300µM), and combination of the two drugs in the presence or absence EGFR activator NSC 228155 (2 µM). Beta-actin was used as loading control.

To establish the mechanisms of Daidzein and Gefitinib combination treatment mediated cell death, we evaluated EGFR signalling pathway and its downstream. Gefitinib is first generation RTKI and works by blocking ATP binding to the tyrosine kinase receptor preventing autophosphorylation and signal transduction 6. Since EGFR can activate many pro-oncogenic and survival signalling pathways, we next examined whether Daidzein and Gefitinib combination treatment would synergistically enhance blockade of EGFR and its downstream. Western blot analysis indeed revealed that Daidzein and Gefitinib combination treatment synergistically inhibited multiple EGFR phosphorylation sites (P-EGFR Y1068, T845 and T1092) in both A549 and H1975 lung cancer cells, which led to deactivation of STAT1 and STAT3 (**Figure 6A**). Inhibition of EGFR also led to downregulation of PI3K, AKT, ERK and interestingly ABCG2, antiporter reported to facilitate development of cancer drug resistance. Additionally, combination treatment upregulated PTEN (**Figure 6B**). To confirm Daidzein and Gefitinib combination treatment blockade of EGFR, we employed nitro-benzoxadiazole (NBD) (NSC 228155) compound which can activate EGFR by directly binding to the dimerization domain of EGFR receptor with a similar phosphorylation profile as the EGF-ligand. NBD treatment led to activation of EGFR at Y1068 phosphorylation site and its downstream, STAT/AKT/ERK (**Figure 6C**). However, when co-treated with Daidzein and Gefitinib combination NBD compound failed to activate EGFR and its downstream effectors. Thus, these data together show that Daidzein and Gefitinib combination treatment enhances EGFR blockade.

### Daidzein and Gefitinib Combination Treatment Synergistically Activates ROS/ASK-1/JNK Pathway in Lung Adenocarcinoma cells

**Figure 7:**
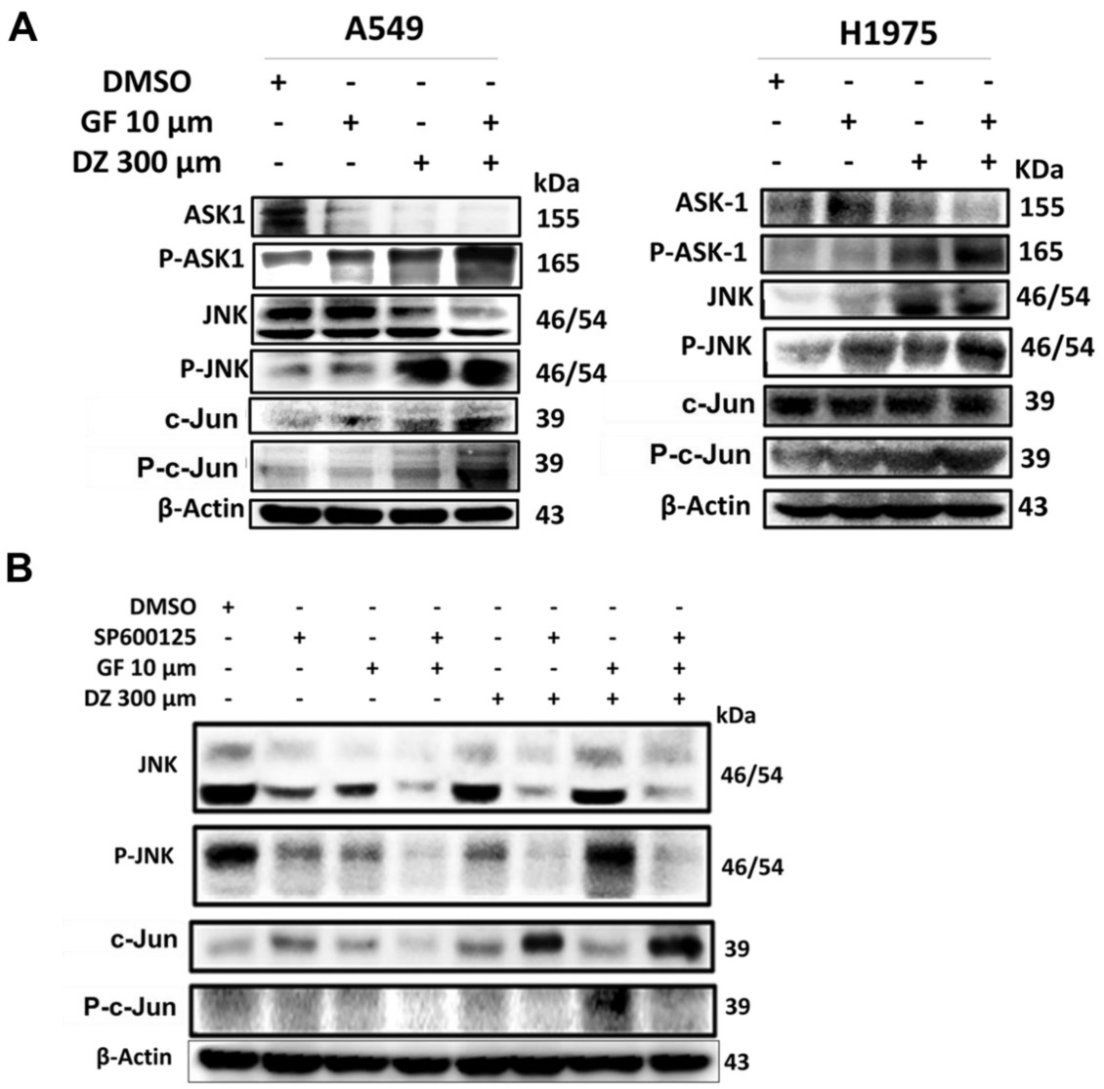
Daidzein and Gefitinib Combination Synergistically activates ASK-1/JNK in Lung Adenocarcinoma cells. Immunoblotting of A549 and H1975 cancer cells whole cell lysates treated with either Gefitinib (10µM), or Daidzein (300µM), and combination of the two drugs. (**A**) Activation of ROS/ASK-1/JNK pathways (**B**) Confirmation of JNK activation, immunoblotting of A549 cancer cells treatment as above in the presence or absence of JNK inhibitor (SP600125 15 µM). Beta-actin was used as loading control.

Previously it has been demonstrated that excessive generation of ROS stress can activate ASK-1 leading to JNK activation and apoptosis 39. We have shown that Daidzein and Gefitinib combination treatment induces ROS and apoptosis (**Figure 3, 4, and 5**). Next, we assessed whether combination treatment inducement of ROS sequentially activated JNK. Indeed, western blot analysis revealed that Daidzein and Gefitinib combination treatment synergistically activated ASK1, which sequentially activated JNK to induce phosphorylation of c-Jun in A549 and H1975 cells (**Figure 7A**). Since JNK plays an important role in apoptotic mediated cell death, we aimed to ascertain if JNK deactivation would influence c-Jun activation and apoptosis induction. We utilized SP600125 (anthrapyrazolone) which inhibits JNK by contending with ATP binding therefore preventing the phosphorylation of c-Jun. Indubitably, co-treatment with SP600125 dramatically reversed the phosphorylation of JNK and c-Jun (**Figure 7B**). Upstream of ROS activation our data revealed as well that co-treatment with NAC reversed JNK phosphorylation and activation (**Figure 5E**). Taken together our data suggests that Daidzein and Gefitinib combination treatment may induce ROS/ASK-1/JNK pathway mediated apoptosis.

### Daidzein and Gefitinib combination treatment Induces Nuclear Localization of c-Jun to Activate Apoptosis

**Figure 8:**
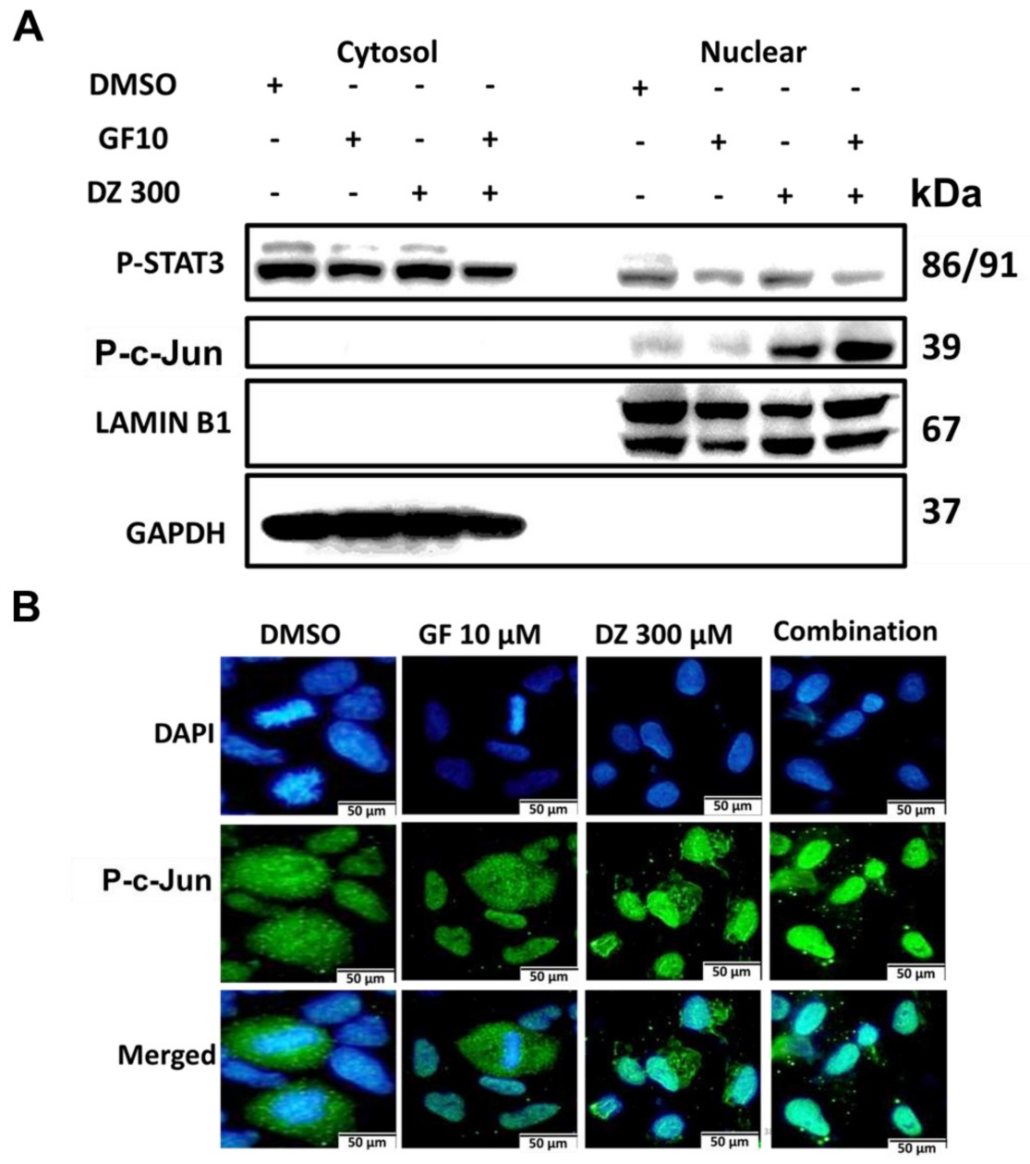
** Daidzein and Gefitinib combination treatment Induces Nuclear Localization of c-Jun to Activate Apoptosis.** (**A**) A549 cancer cells were treated with either Gefitinib (10 µm) or Daidzein (300 µm) and combination of the two drugs followed by nuclear and cytosolic protein fractionation and immunoblotting of phosphorylated STAT3 and c-Jun in A549 cancer. GAPDH and LAMIN B1 were used as a loading control of cytosolic and nuclear proteins respectively. (**B**) A549 cancer cells grown on glass slides were treated with either Gefitinib (10 µm) or Daidzein (300µm) and combination of the two drugs followed by immunofluorescence staining of phosphorylated c-Jun. Representative images are shown. Scale bars: 50 μm.

Our results show that Gefitinib and Daidzein combination induces ROS (**Figure 3 and 5D**) which led to activation of JNK and c-Jun (**Figure 7E**). We next investigated whether activation of JNK led to nuclear translocation of c-Jun. Western blot analysis of nuclear and cytosol fractionated proteins revealed that Daidzein and Gefitinib combination treatment significantly increased expression of phosphorylated c-Jun in the nuclear protein fraction, concomitantly suppressing the expression of p-STAT3 (**Figure 8A**). Immunofluorescence staining of phosphorylated c-Jun confirmed its nuclear accumulation (**Figure 8B**). Taken together, our data suggests that Daidzein and Gefitinib combination treatment synergistically induces nuclear translocation of phosphorated c-Jun to activate apoptosis.

### Daidzein and Gefitinib Combination treatment induces Sub G1 Accumulation and G0/G1 Cell Cycle Arrest

**Figure 9:**
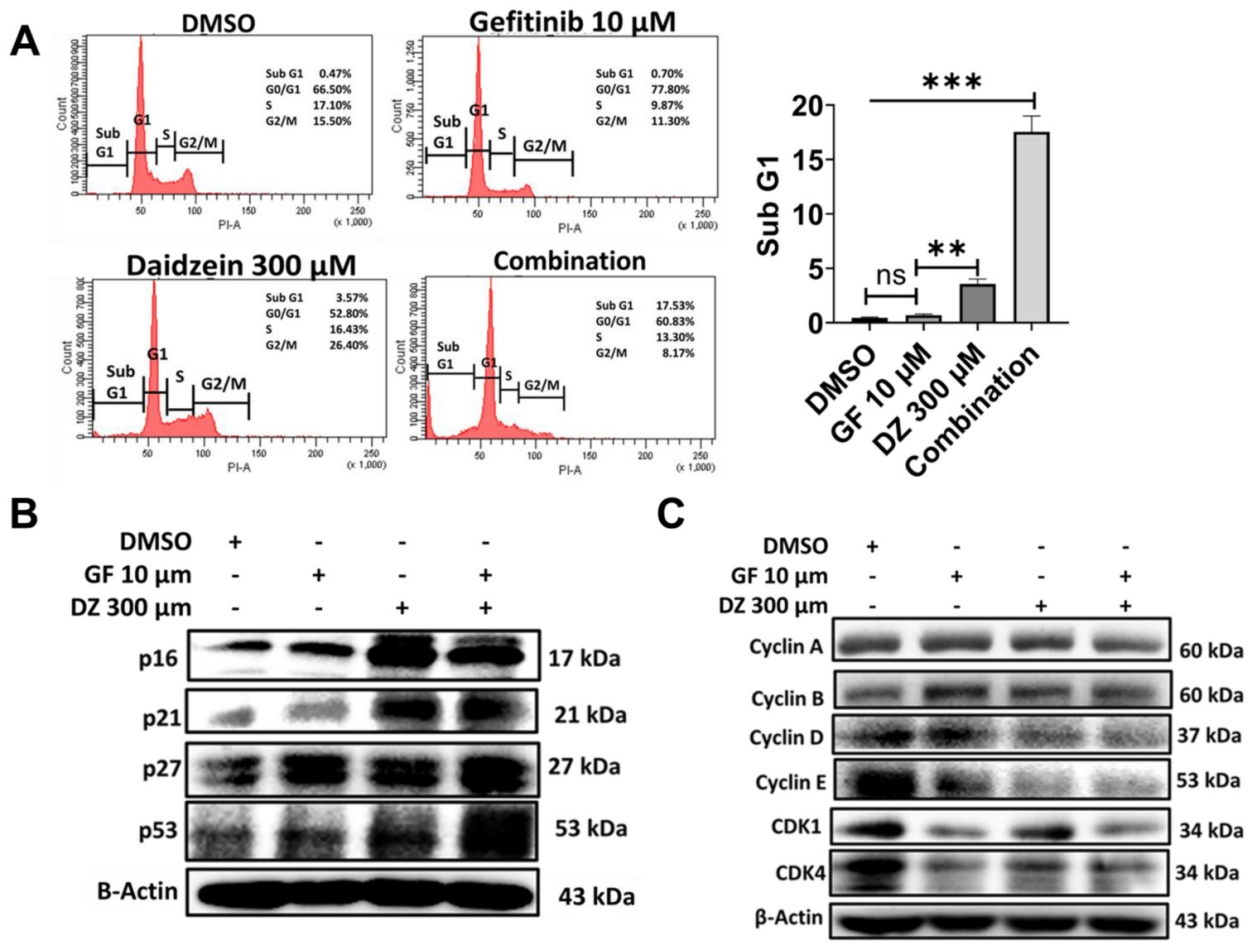
** Daidzein and Gefitinib Combination treatment induces Sub G1 Accumulation and G0/G1 Cell Cycle Arrest.** (**A**) Cell cycle flow cytometric analysis, A549 cancer cells were treated with Gefitinib (10µM), or Daidzein (300µM), or the combination of the two drugs. The cells were stained with PI followed by flow cytometry and quantification of the sub-G1 fraction of the cell cycle. (**B** and **C**) Same treatment as in A, Immunoblot of cell cycle markers in A549 cancer cells whole cell lysates. Beta-actin was used as a loading control. Results are shown as means ± standard deviation from three independent experiments. ns: not significant, **P<0.001, ***P<0.0001

Growth inhibition and apoptosis are often preceded by halt of cell cycle progress. To examine whether Gefitinib and Daidzein combination treatment induces arrest of cell cycle, we performed propidium iodide (PI) DNA staining and cytometric analysis. Our data revealed that Gefitinib and Daidzein combination treatment induced apoptotic Sub G1 peak (**Figure 9A**). A549 cancer cells in G1 phase were 78% when treated with 10 µM Gefitinib, 53% when treated with Daidzein 300 µM and 61% when treated with combination of the two drugs compared to 67% of DMSO control group (**Figure 9A**). We then next assessed western blot analysis of G1 phase related proteins. Our results reveled that Daidzein and Gefitinib combination treatment significantly upregulated the expression levels of p53, and its downstream targets p27, p21, and p16 (**Figure 9B**), which led to inhibition of cyclin D1 and cyclin E (**Figure 9C**). These results suggest Daidzein and Gefitinib combination treatment induces subG1 accumulation and a G0/G1 cell cycle arrest.

### Daidzein and Gefitinib combination treatment significantly suppresses A549 lung cancer cells in nude mice tumor xenograft

**Figure 10:**
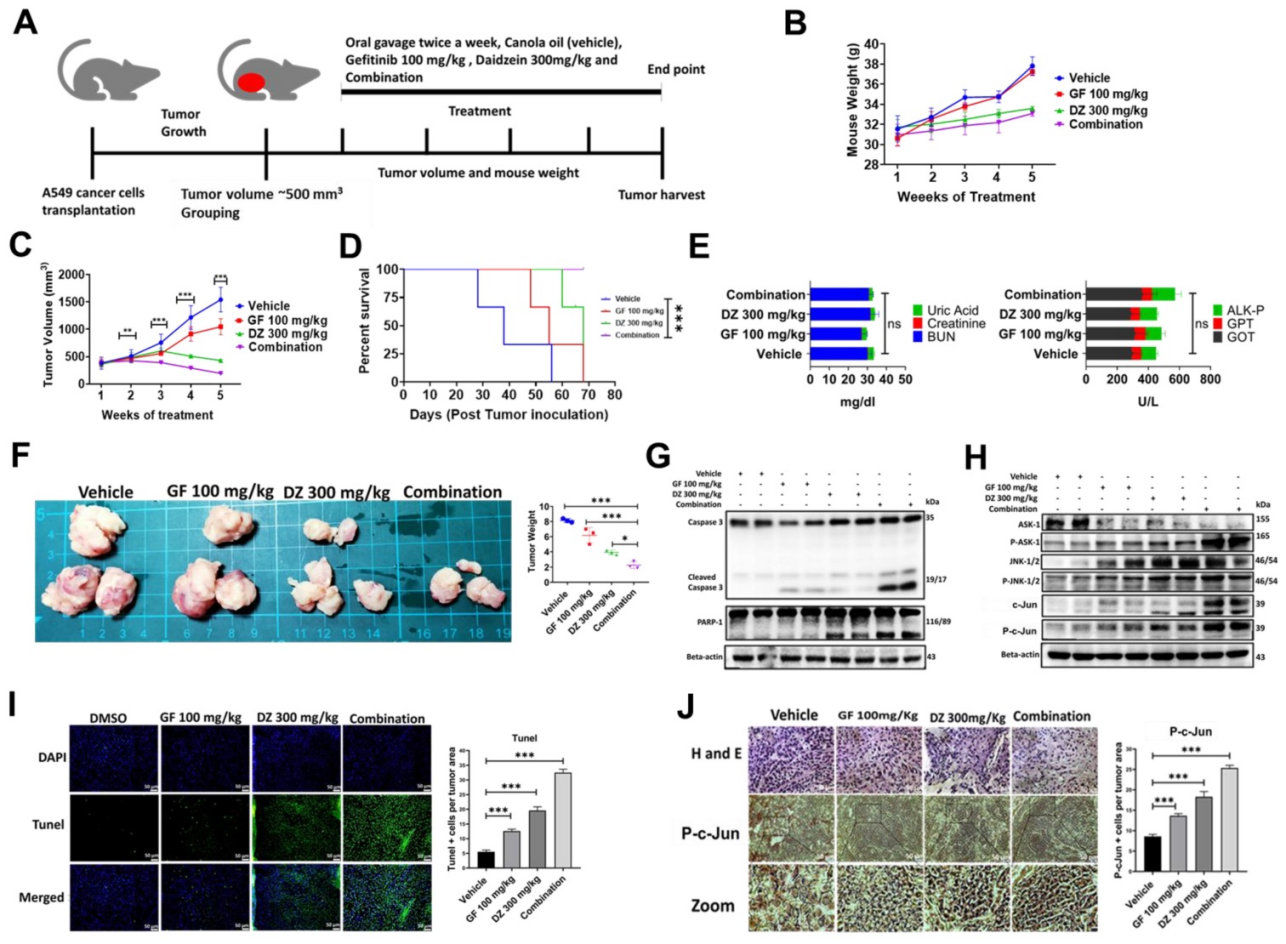
** Daidzein and Gefitinib combination treatment significantly suppresses A549 lung cancer cells in nude mice tumor xenograft.** (**A**) Combination treatment experimental design. (**B**) Monitoring of mice body weight during the treatment period. (**C**) Tumor volume quantification of established A549 tumors in NU/NU mice treated with vehicle, Gefitinib, Daidzein, or combination of the two drugs (**D**) Kaplan-Meier survival curve of A549 lung adenocarcinoma tumor-bearing mice following tumor initiation to the end point of the experiment. (**E**) Determination of GPT and GOT for liver function and BUN, Creatine and Uric Acid for kidney function combination treatment safety evaluation. (**F**) Tumors harvested at the end of the experimental period and quantification of their respective weights. (**G** and **F**) Immunoblot of A549 cancer cells xenografted tissue whole cell lysates. Beta-actin was used as loading control. (**I**) Staining and quantification of TUNEL-positive cells in tumor sections from the different treatment groups harvested at the endpoint. Representative images are shown. Scale bars: 50 μm. (**J**) H&E, and immunostaining of phosphorylated c-Jun in A549 cancer cells tumor tissue sections. Representative images are shown. Scale bars: 50 μm. Results are shown as means ± standard deviation from three independent experiments. *P < 0.05; **P < 0.001; ***P < 0.0001, log-rank test (D).

Our in vitro data has demonstrated that daidzein and gefitinib combination treatment synergistically inhibited the growth of A549 and H1975 Lung adenocarcinoma cells. We next explored the antitumor effects of Daidzein and Gefitinib combination treatment in lung cancer tumor xenografts. A549 cancer cells were transplanted into sides of NU/NU mice. The mice were randomly grouped into four groups when the tumor volume was approximately 500 mm^3^ (three mice per group). The mice were treated through oral gavage with either Gefitinib 100 mg/kg, or Daidzein 300 mg/kg, and the combination of the two the drugs suspended in canola oil. the control group were given only canola oil (**Figure 10A**). During the treatment period the mice showed neither weight loss nor drug induced toxicity (**Figure 10B**). Importantly, Daidzein and Gefitinib combination significantly reduced tumor volume than Gefitinib alone or Daidzein alone compared to the control group (**Figure 10C**). Furthermore, Kaplan-Meier survival estimation revealed that mice in combination treatment group had a longer survival compared those treated with single drugs alone (**Figure 10D**). The mice were euthanized after a five-week treatment period. The tumors, whole blood and internal organs were collected. Gross examination of internal organs of all animals revealed no abnormalities, and liver and kidney function test analysed on the blood serum demonstrated no significant differences between the treatment groups (**Figure 10E**). Outstandingly, combination treatment reduced gross tumor weights and sizes compared to vehicle and single drugs alone (**Figure 10F**). Western blot analysis of whole cell tissue lysates confirmed induction of apoptosis through the ASK-1/JNK pathway, cleavage of caspase 3, PARP-1 (**Figure 10G and H**). Tunel staining on tumor tissue confirmed that the combination treatment induced more Tunel positive apoptotic cells than single drugs alone (**Figure 10I**). Finally, tumor tissue staining of phosphorylated c-Jun confirmed increased nuclear accumulation of p-c-Jun in the combination treatment group compared to Daidzein alone and Gefitinib alone (**Figure 10J**). Taken together, these data show that Daidzein and Gefitinib combination treatment in vivo exhibited potent tumor reduction synergism.

## Discussion

**Figure 11:**
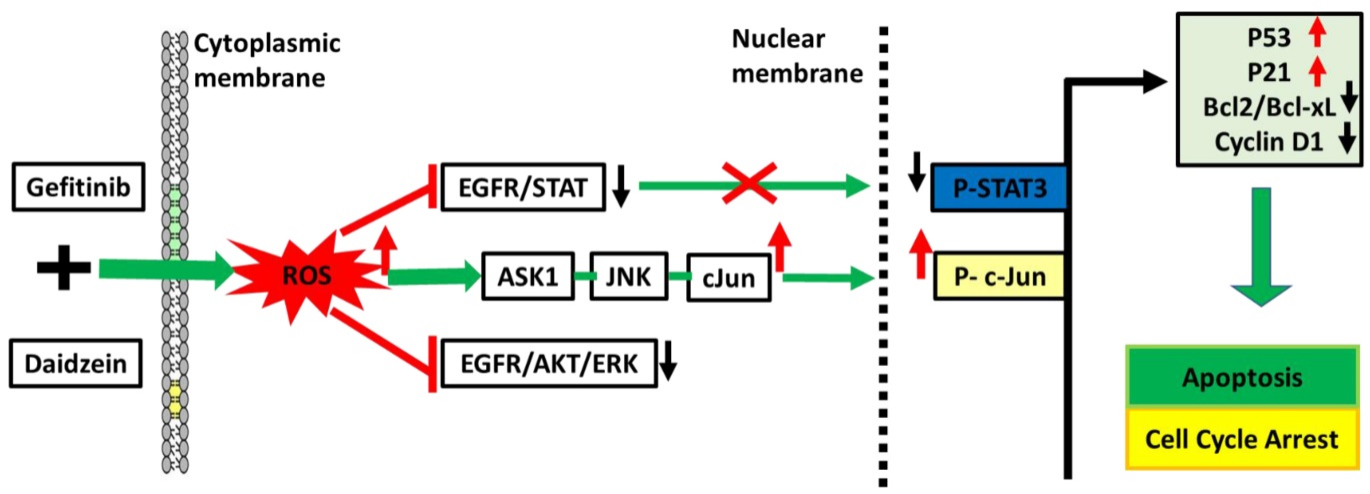
Schematic pathway induced by Daidzein and Gefitinib combination treatment

Daidzein has been shown in previous studies to have a wide range of bioactivities 9-15, 40-43. Here, we have demonstrated Daidzein's select cytotoxicity and safeness in normal cells and cancer cells (**Figure 1**). We have also demonstrated Daidzein and Gefitinib combination treatment potent synergistic cytotoxicity against lung adenocarcinoma cells (**Figure 2**)**.** We have shown that Daidzein and Gefitinib combination treatment synergistically induced persistent ROS/ASK1/JNK activation, and c-Jun nuclear translocation. Inhibition of EGFR-STAT/AKT/ERK signalling pathways, a G0/G1 cell cycle arrest and apoptosis in lung cancer cells (**Figure 11**).

ROS is recognized as an important second messenger upstream of survival and cell death pathways. Physiological levels of ROS regulate cellular metabolism, while excessive ROS can induce cell death 44, thus, selective ROS induction in cancer cells can be a druggable target in the development of anticancer drugs. In this present study, we demonstrate that Daidzein and Gefitinib combination treatment synergistically induced ROS generation (**Figure 3**). Generation of ROS was remarkedly reduced when co-treated with a NAC antioxidant (**Figure 4D**). Thus, generation of ROS may be a critical anticancer mechanism of Daidzein and Gefitinib combination treatment. ASK1/MAPK signaling pathways under ROS stress promotes apoptosis through p38 and JNK 32, 45. Our results show that ROS stress induced by Daidzein and Gefitinib combination treatment synergistically activated ASK1 which in turn increased JNK phosphorylation and c-Jun translocation to the nucleus to induce apoptosis (**Figure 7**, **Figure 8**). JNK inhibitor SP600125 markedly reduced phosphorylation and activation of JNK and c-Jun, suggesting synergism of Daidzein and Gefitinib combination treatment contributed to JNK activation and apoptotic cell death (**Figure 7 C**).

Apoptosis is important in the early development and tissue homeostasis maintenance. Dysregulated apoptosis is associated with tumor development 46. Our results demonstrates that Daidzein and Gefitinib combination treatment induced both intrinsic and extrinsic apoptosis of lung cancer cells (**Figure 4 and 5**). Bcl2 family members are crucial regulators of cell death or survival depending on the presence of stress or survival signals 47. In response to growth factors, protein kinases such as AKT phosphorylate Bcl-2 family members to induce growth and proliferation. It has been previously demonstrated that AKT phosphorylates Bad at Ser-112 and Ser-136 to inhibit cell death. When BAD become phosphorylated at S112 and S136 it translocates to the cytosol bound to 14-3-3 proteins there by releasing its inhibitory effect on Bcl-2 and Bcl-XL ensuring cell survival 48. Bad induces cell death in the absence of phosphorylation at these sites, perhaps through heterodimerization with Bcl2 and Bcl-xL and a concomitant homodimerization with Bax 49. Similarly, here we show that Daidzein and Gefitinib combination treatment synergistically reduced the expression of AKT (**Figure 6A**), which led to downregulation of phosphorylated Bcl2 and Bad at Ser112 and upregulation Bax to induce apoptosis, which was confirmed by Tunel and annexin V-FITC and PI double staining (**Figure 4F and E, Figure 5 C**).

Cell cycle progress is directly associated with tumor proliferation and growth, hence targeting cell cycle proteins in the novel anticancer drugs discovery is generating a lot of interest 50. Here we show that Daidzein and Gefitinib combination treatment synergistically induced accumulation of subG1 apoptotic cell population and a G0/G1 phase cell cycle arrest (**Figure 9**). Cell cycle is regulated by balance of cyclins and cyclin-dependent kinases (CDKs), inhibitory factors such as p27, p21, and p16 51. In our study, we reveal that Daidzein and Gefitinib combination treatment synergistically upregulated expression of p53, p27, p21, and p16, while downregulating cyclin D1 and E (**Figure 9B and C**). G1 phase is controlled by expression levels of cyclin D1, p21 and p27. Since p53 plays important role in cell cycle regulation by controlling levels of p21 and p27, it was assumed that excessive production of ROS by Daidzein and Gefitinib combination treatment induced a G0/G1 cell cycle arrest by increasing the levels of p53, thereby influencing the levels of p21 and p27 and hence cyclin D1 expression.

Previously in vivo studies have demonstrated Gefitinib weekly dose of 400 mg/kg was less toxic than 80mg/kg daily dose in mouse lung cancer model 26 and Daidzein daily oral doses of 250mg/kg and 1000 mg/kg did not cause toxicity in female rats 52. Similarly, in our present animal study, no apparent weight loss or toxicity was observed in the NU/NU mice. After sacrificing the animals at the end of treatment period, analysis of serum liver and kidney toxicity markers demonstrated no significance differences between all the treatment groups (**Figure 10B and E**). During the treatment period combination treatment remarkably reduced the tumor volume than Daidzein alone or Gefitinib alone compared to the vehicle control and thus translating to a longer survival on Kaplan-Meier survival analysis (**Figure 10 C and D**). Importantly, at the experimental endpoint combination treatment significantly repressed tumor growth as shown by small tumor size and weights than single drugs alone compared to the control vehicle. Taken together all these data confirms Daidzein and Gefitinib combination synergistical potential in the treatment of lung adenocarcinoma.

In conclusion, our in vitro and in vivo studies have demonstrated that Daidzein may enhance Gefitinib chemotherapeutic efficacy. By reducing the clinical dose of Gefitinib, and the minimal toxicity observed in both in vitro and in vivo experimental models shows that our treatment strategy may reduce side effects associated with chemotherapy toxicity. Daidzein supplements with current chemotherapeutic agents may well be an alternative strategy to improve the treatment efficacy of lung adenocarcinoma.

## Supplementary Material

Supplementary figures.

## Figures and Tables

**Table 1:** IC_50_ values for Daidzein and Gefitinib treated against A549, H1975 and LOVO cancer cells for the indicated times

		Std. Deviation	95% CI	
Cell/Treatment	Mean	(±)	Lower	Upper
A549 Daidzein 24 h	226.2	1.2	223.3	229.1
A549 Daidzein 48 h	130.5	1.6	126.6	134.4
H1975 Daidzein 24 h	257.3	1.1	254.5	260.1
H1975 Daidzein 48 h	186.5	1.1	183.8	189.1
LOVO Daidzein 24 h	249.2	1.3	246	252.3
A549 Gefitinib 48 h	22.8	0.4	21.9	23.7
H1975 Gefitinib 48 h	21.7	1.0	19.2	24.3
