# Supplementary material for: Daidzein Synergizes with Gefitinib to Induce ROS/JNK/c-Jun Activation and Inhibit EGFR-STAT/AKT/ERK Pathways to enhance Lung Adenocarcinoma cells chemosensitivity
Source: Int J Biol Sci. 2022 May 16;18(9):3636–52. doi: 10.7150/ijbs.71870 (PMC9254481; doi:10.7150/ijbs.71870)
Supplement: Supplementary file 1 — Supplementary figures. [file ijbsv18p3636s1.pdf]

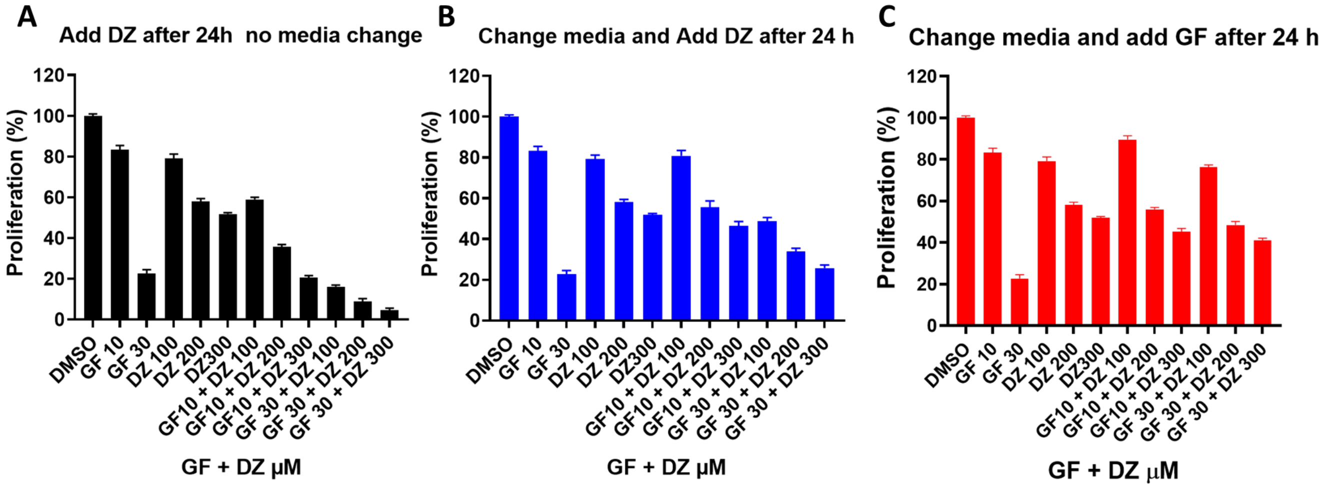

Supplementary figure 1: Evaluation of Daidzein and Gefitinib combination treatment potential

(A) A549 cancer cells were co-treated first with either 10 µM or 30 µM of Gefitinib and the indicated concentrations of Daidzein were added after 24 hours without changing media followed by MTT assay cell viability evaluation. **(B)** As in **A;** A549 cancer cells were treated with Gefitinib and then the indicated concentrations of Daidzein were added after changing media. **(C)** As in **A;** A549 cancer cells were treated with Daidzein and the indicated concentrations of Gefitinib were added after changing media. These results are expressed as a percentage of viable cells from treated groups compared with control cells treated with DMSO.

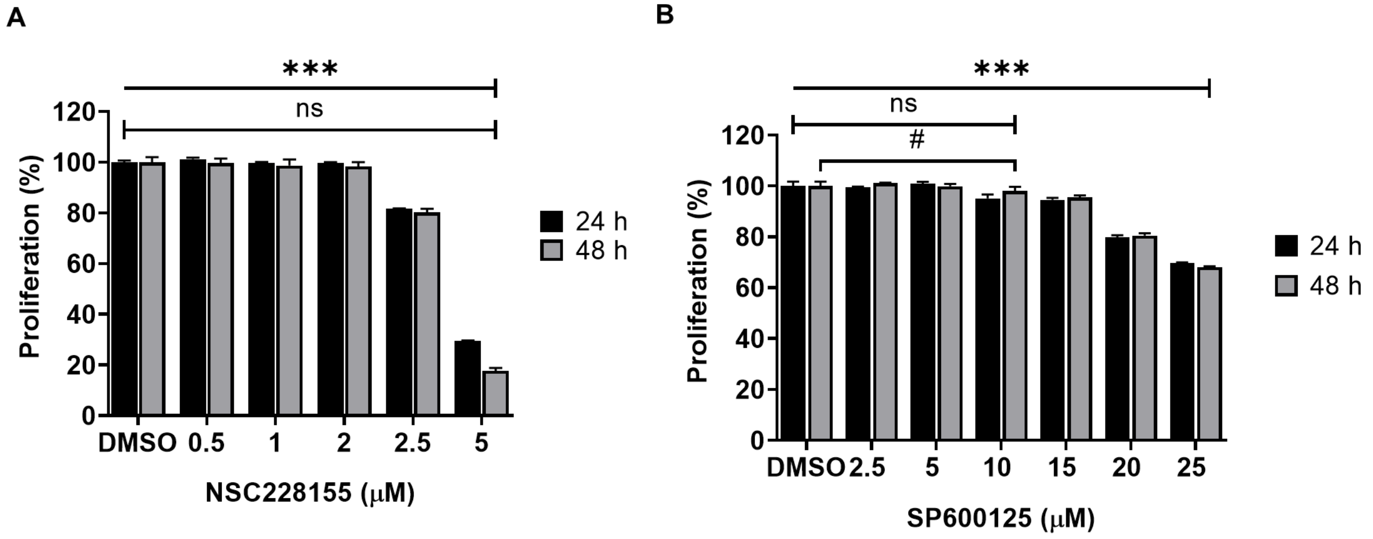

Supplementary Figure 2: Influence of NSC228155 (EGFR Activator) and SP600125 (JNK inhibitor) on A549 cancer cells

(A) The effect of NSC228155 on A549 cancer cells treated with the indicated concentrations, cell viability was evaluated by MTT assay. **(B)** The influence of SP600125 on A549 cancer cells, treated with indicated concentrations. These results are expressed as a percentage of viable cells from treated groups compared with control cells treated with DMSO. The results are shown as means ± standard deviation from three independent experiments. ns: not significant, # $p < 0.05$, *** $p < 0.0001$.
